# Ten years of genome‐wide association studies of immune‐related diseases

**DOI:** 10.1002/cti2.1022

**Published:** 2018-06-15

**Authors:** Manuel AR Ferreira

**Affiliations:** ^1^ QIMR Berghofer Medical Research Institute Brisbane QLD 4029 Australia

Genome‐wide association studies (GWAS) represent the gold standard approach to identify common genetic variants that explain variation in human quantitative traits and disease risk. It is now just over 10 years since this approach was first applied to immune‐related diseases.^1^, ^2^ To mark this milestone, this Special Feature of CTI includes reviews that summarise the key findings from published GWAS of five immune‐related diseases, namely ankylosing spondylitis (AS),^3^ asthma,^4^ Crohn's disease (CD),^5^ multiple sclerosis (MS) 6 and type 1 diabetes (T1D).^7^

In the last 10 years, GWAS collectively identified 39 independent associations for asthma, 54 for T1D, 113 for AS, 233 for MS and 241 for CD. A key factor explaining this difference in number of reported associations is GWAS sample size but other factors are also important, some discussed by Vicente *et al:* 4 disease prevalence, genetic architecture and genotyping array and/or haplotype reference panel used for imputation. Despite this difference in yield between diseases, all five reviews highlight a now widely accepted view that GWAS, combined with the use of a significance threshold that appropriately accounts for multiple testing, revolutionised our ability to identify genetic associations that are reproducible, which was generally not the case with previous approaches (i.e. linkage analysis and candidate gene studies).

What have we learnt from results of these GWAS? For all five diseases, results from GWAS confirmed strong associations with genetic variants located in the major histocompatibility complex (MHC), which had been previously identified as a major risk locus for many immune‐related diseases by linkage and/or candidate gene studies. Specific associations at the MHC are discussed in detail by Li and Brown for AS,^3^ Cotsapas and Mitrovic^6^ for MS and Pociot for T1D.^7^ However, as Pociot highlights, the biological mechanisms underlying the MHC associations with immune‐related conditions remain very poorly characterised. This is a consequence of the complex LD patterns in the region, as well as the high density of genes and genetic variants present, including HLA alleles. This complexity makes this a difficult but not intractable region for functional studies, as elegantly shown recently for schizophrenia.^8^

Of note, most risk variants reported in GWAS of immune‐related diseases are not located in the MHC. Importantly, a small number of these non‐MHC associations have already led to an improved understanding of disease pathophysiology. These include the discovery that autophagy, IL‐17/IL‐23 signalling and type 3 innate lymphoid cells play an important role in Crohn's disease (discussed by Verstockt *et al*.^5^) and that variation in the ability to trim peptides for presentation by HLA class 1 molecules is associated with AS risk (discussed by Li and Brown^3^). Other novel insights included the identification of cell types that are likely to mediate the effect of genetic variants on disease risk, which include CD4^+^ T‐cell subsets for MS, T1D and asthma, as well as the discovery that there is a substantial overlap in genetic risk factors between seemingly distinct immune‐related diseases. The latter finding can be exploited to identify associations that are shared across diseases, as was the case for some GWAS reviewed by Li and Brown^3^ and Vicente *et al*.^4^ However, it is now also clear that not all shared genetic associations have the same directional effect across different diseases: that is, the same allele might be associated with higher risk of one disease but lower risk of another disease. This directional information might be useful to determine whether a biological therapy that targets the gene underlying a shared genetic association is likely to be effective across different immune diseases, as highlighted by Verstockt *et al*.^5^ and Vicente *et al*.^4^

The ability of results from GWAS to inform disease mechanisms depends, to a large extent, on our ability to understand the functional consequences of genetic variants. This is not an easy task, as highlighted by all five reviews, and is considered to be one of the major bottlenecks preventing (or slowing down) the translation of GWAS findings into novel targeted therapies. Specifically, risk variants are highly enriched amongst intronic or intergenic regulatory elements, and so most are expected to affect gene transcription and not protein sequence. Information from expression quantitative trait loci (eQTL) can be leveraged to identify the likely target genes, as done systematically for all published asthma risk variants by Vicente *et al*.^4^ However, this approach has limitations, as pointed out by Vicente *et al*.^4^ and Cotsapas and Mitrovic,^6^ and so the predicted target genes should be validated functionally, which can be time‐consuming.

Having identified genes that are the targets of disease‐associated variants, it becomes important to determine whether such genes represent promising drug targets. This requires studying gene function, which is particularly challenging for genes that are poorly characterised, including non‐coding RNAs. In this context, appropriate high‐throughput assays of immune cell function are essential, as are animal studies. In contrast, when the function of a target gene is well characterised in the literature, then this greatly facilitates the design of animal studies and, ultimately, clinical trials. An example of this is the IL‐6 receptor, discussed by Vicente *et al.,* 4 which is the target of biological therapies approved for rheumatoid arthritis which could potentially be repurposed to treat asthma. Other examples of genes that are the likely targets of risk variants and that are being considered for clinical development include *ADORA1*,* TLSP* and *IL1RL1* for asthma, and *IL‐23* and related genes for AS.

Looking ahead at what might be expected from GWAS in the next 10 years, all five reviews highlight as a priority understanding how genetic variation contributes to differences in clinical presentation, including age of onset, progression and severity. This can be achieved by applying the GWAS approach to study variation in clinical parameters within affected individuals, which should be very fruitful provided that reliable phenotypes can be measured in large sample sizes. Other underexplored areas that are highlighted as priorities include understanding the extent to which gene‐by‐gene, gene‐by‐environment and epigenetic effects contribute to disease risk; testing regions of the genome that have been largely ignored in previous GWAS (e.g. X chromosome, genes from the killer‐cell immunoglobulin‐like receptor (KIR) family); and identifying risk factors in non‐European populations.

In conclusion, in the last 10 years GWAS have discovered hundreds of reproducible genetic associations with common immune‐related conditions. The reviews in this Special Feature of *Clinical & Translational Immunology* highlight that these findings are now slowly but steadily helping us understand the underlying disease biology in greater detail.

## Conflict of interest

The author declares no conflict of interests.
